# Longitudinal COVID-19 Vaccination Records and SARS-CoV-2 Infection Among Older Long-Term Care Facility Residents in Okinawa, Japan: A Retrospective Descriptive Study

**DOI:** 10.7759/cureus.115946

**Published:** 2026-09-08

**Authors:** Norifumi Kudeken

**Affiliations:** 1 Internal Medicine, Kubagawa Medical Clinic, Naha, JPN

**Keywords:** breakthrough infection, covid-19, long-term care facility, older adults, reinfection, sars-cov-2, vaccination

## Abstract

Background

Repeated COVID-19 vaccination is widely used in long-term care facilities, yet SARS-CoV-2 infection and reinfection continue to occur in frail older populations. Resident-level longitudinal data linking vaccination records with first and recurrent infections remain limited in Japanese long-term care settings.

Materials and methods

We conducted a retrospective descriptive study in a 134-bed long-term care facility in Okinawa, Japan. We linked 976 valid, dated vaccination records for 232 residents from June 7, 2021, through January 16, 2026, to 136 infection episodes among 109 residents from May 22, 2023, through April 30, 2025, using de-identified resident identifiers. Because facility occupancy was dynamic and complete admission and discharge dates were unavailable, not all 232 residents were continuously at risk. Recurrent infection was operationally defined as a new symptomatic episode with a new positive antigen test at least 60 days after the preceding episode, following documented recovery and release from isolation. Analyses were descriptive; incidence rates, vaccine effectiveness, and inferential statistics were not estimated.

Results

Among 232 residents, 157/232 (67.7%) were female. The mean age at first recorded vaccination was 85.5 ± 8.3 years, and the median total number of documented doses was 4 (interquartile range [IQR], 2-7). Linkage was successful for 89 residents with 114 infection episodes; 20 residents with 22 episodes could not be linked. Linked documented infection occurred in 89/232 residents (38.4%; 95% confidence interval [CI], 32.3%-44.8%). Among these 89 residents, 65/89 (73.0%) had one episode, 23/89 (25.8%) had two, and 1/89 (1.1%) had three. Recurrent infection occurred in 24/89 residents (27.0%; 95% CI, 18.8%-37.0%). The median interval from the first to second episode was 364 days (IQR, 289.5-575; range, 286-599). Before first infection, the median number of documented doses was 5 (IQR, 2-6; range, 0-8). Among 84 residents with prior documented vaccination, the median interval from the most recent dose to first infection was 49 days (IQR, 14.5-131; range, 3-488). Eight aggregate hospitalizations were documented during infection surveillance but could not be linked to the vaccination roster; one resident died during hospitalization and seven returned to the facility.

Conclusions

Linking longitudinal vaccination and infection records was feasible and demonstrated a continuing burden of infection and reinfection. However, dynamic occupancy and incomplete time-at-risk data precluded incidence, person-time, dose-specific vaccine-effectiveness, or causal analyses. The dose distribution should not be interpreted as evidence that additional vaccination increased infection risk. Long-term care facilities require layered prevention combining vaccination with rapid testing, infection-control measures, and timely clinical management.

## Introduction

Residents of long-term care facilities remain at high risk during COVID-19 outbreaks because of advanced age, multimorbidity, functional dependence, close-contact living conditions, and the difficulty of complete physical separation within shared residential environments [1-3]. Repeated vaccination has reduced severe outcomes in older adults, but protection against infection is incomplete and may diminish as time from vaccination increases and new variants emerge [4-7].

Japanese reports have described COVID-19 outbreaks and prevention strategies in elderly care facilities [8,9]. However, longitudinal resident-level data linking repeated vaccination histories with subsequent infection and reinfection are still limited. Such linkage is important because a simple count of infections or vaccine doses cannot distinguish descriptive patterns from vaccine effectiveness.

A previous report from the same facility examined clinical outcomes among 107 residents with mild COVID-19 who received early molnupiravir [10]. The present study addresses a different research question: the linkage of a multi-year vaccination roster to first and recurrent SARS-CoV-2 infections among facility residents. We aimed to describe the vaccination roster, the cumulative proportion with documented COVID-19, recurrent infection, and the distribution of doses received before first infection without estimating dose-specific vaccine effectiveness.

## Materials and methods

Study design and setting

This retrospective descriptive study was conducted in a 134-bed long-term care facility in Okinawa, Japan, served by Kubagawa Medical Clinic. The facility provides care for frail older adults with substantial functional dependence. Routinely maintained vaccination and COVID-19 records were reviewed and linked using de-identified resident identifiers.

Concurrent facility measures during documented COVID-19 outbreaks included symptom and temperature surveillance, antigen testing, isolation and in-place cohorting, use of personal protective equipment, and natural ventilation by opening windows. Positive test results were communicated to the attending physician, clinically indicated antiviral treatment was arranged, and residents requiring hospital-level care were transferred. These measures were reported as contextual features of facility practice and were not evaluated as independent exposures or interventions. No additional topical or pharmacological prophylactic regimens were implemented as institutional measures.

Data sources and study periods

The vaccination dataset covered June 7, 2021, through January 16, 2026. After exclusion of five non-data worksheet rows, the cleaned vaccination roster contained 976 valid, dated vaccine-dose records among 232 residents. Documented vaccines were COVID-19 mRNA vaccine products administered across four formulation periods: original/early-period, bivalent ready-to-use, XBB.1.5 ready-to-use, and the subsequent syringe formulation for persons aged 12 years or older. Product-neutral terminology was used because the study was not designed to compare manufacturers or formulations.

Facility COVID-19 records covered May 22, 2023, through April 30, 2025, and contained 136 documented infection episodes among 109 residents. Diagnoses were made by antigen testing. Records were linked using de-identified resident identifiers. Eighty-nine residents with 114 infection episodes were linked successfully to the vaccination roster. Twenty infected residents with 22 episodes could not be linked and were excluded from vaccination-linked analyses. Diabetes status was obtained from a facility-wide clinical list, and residents receiving insulin were identified separately.

The 232 residents were identified from the longitudinal vaccination roster; however, because facility occupancy changed over time, not all residents were continuously present at the facility or under observation throughout the study period. Facility occupancy changed because of admissions, discharges, hospitalizations, and deaths, and complete admission and discharge dates could not be reconstructed. Annual health-screening rosters contained 127 residents on December 6, 2022, 135 on December 5, 2023, and 117 on December 10, 2024. These counts were treated only as cross-sectional reference snapshots and were not used as outbreak-specific denominators or estimates of the population at risk.

Outcomes and definitions

The primary descriptive outcome was linked documented COVID-19 among residents represented in the vaccination roster. Residents without a linked infection record were classified as having no linked documented infection and were not assumed to have remained uninfected throughout follow-up.

For the revised analysis, recurrent infection was operationally defined as a new symptomatic episode with a new positive antigen test at least 60 days after a preceding documented episode, following documented clinical recovery and release from isolation. This definition was formalized during manuscript revision rather than prospectively prespecified. A sensitivity description using a minimum interval of 90 days was also performed. No viral sequencing was available; therefore, no individual infection episode was attributed to a specific SARS-CoV-2 variant.

For residents with linked infection records, we determined the number of documented infection episodes per resident, the interval between consecutive episodes, the number of documented vaccine doses before first infection, and the interval between the most recent documented vaccination and first infection. Infection episodes were grouped into six facility outbreaks and three sporadic calendar periods. The most recent vaccine formulation recorded before each episode was classified descriptively. Exact numbers of residents at risk during individual periods were unavailable because daily occupancy and complete entry and exit dates could not be reconstructed.

Available facility records were also reviewed descriptively for hospitalization, oxygen requirement, antiviral treatment, and mortality. However, individual hospitalization, oxygen-treatment, and antiviral-treatment records could not be linked systematically to the vaccination and recurrent-infection datasets. Oxygen administered at the facility was used only as temporary stabilization before hospital transfer for residents with SpO_2_ ≤ 93%; no resident continued living at the facility while receiving ongoing oxygen therapy.

Relationship to the previous publication

The previously published article from this facility focused on early antiviral treatment and clinical outcomes in 107 residents with mild COVID-19 during an overlapping period [10]. The present analysis uses a distinct vaccination roster of 232 residents and 976 documented doses and evaluates resident-level linkage with first and recurrent infections. Treatment response, hospitalization, and mortality from the previous cohort were not reused as resident-level outcomes in the present vaccination-linked analysis. The previous publication is cited to clarify the partial overlap in facility setting and infection-record periods.

Statistical analysis

Analyses were descriptive. Categorical variables are reported as n/N (%) in the text and as n (%) in tables with explicitly stated column denominators. Wilson 95% confidence intervals (CIs) were calculated for the principal proportions, including the proportion with linked documented infection and the proportion with recurrent infection. Continuous variables are summarized using the mean and standard deviation or the median, interquartile range (IQR), and range, as appropriate.

Because complete admission, discharge, and continuous time-at-risk data were unavailable, incidence rates, attack rates, person-time analyses, time-varying analyses, and vaccine-effectiveness estimates were not calculated. The number of doses documented among infected residents was treated as vaccination history and not as a dose-specific infection rate. No inferential statistical tests were performed; therefore, no test statistics, p-values, or statistical-significance threshold were applicable. No multivariable or causal analysis was performed.

Ethical considerations

The study protocol was approved by the Kubagawa Medical Clinic Institutional Review Board (KM-2026-01). The requirement for individual informed consent was waived because the study used de-identified, routinely collected records and involved no intervention.

## Results

Vaccination roster

The vaccination roster included 232 residents and 976 documented COVID-19 vaccine doses. The mean age at first recorded vaccination was 85.5 ± 8.3 years, 157 residents (67.7%) were female, and the median number of documented doses per resident was 4 (IQR, 2-7). Diabetes was documented in 17/232 residents (7.3%), and 2/232 residents (0.9%) received insulin.

The infection dataset contained 136 documented episodes among 109 residents. Eighty-nine residents with 114 infection episodes were successfully linked to the vaccination roster, whereas 20 residents with 22 episodes could not be linked. Linked documented COVID-19 occurred in 89/232 residents (38.4%; 95% CI, 32.3%-44.8%). The remaining 143/232 residents (61.6%) were classified as having no linked documented infection and were not assumed to have remained uninfected throughout the observation period (Figure 1).

**Figure 1:**
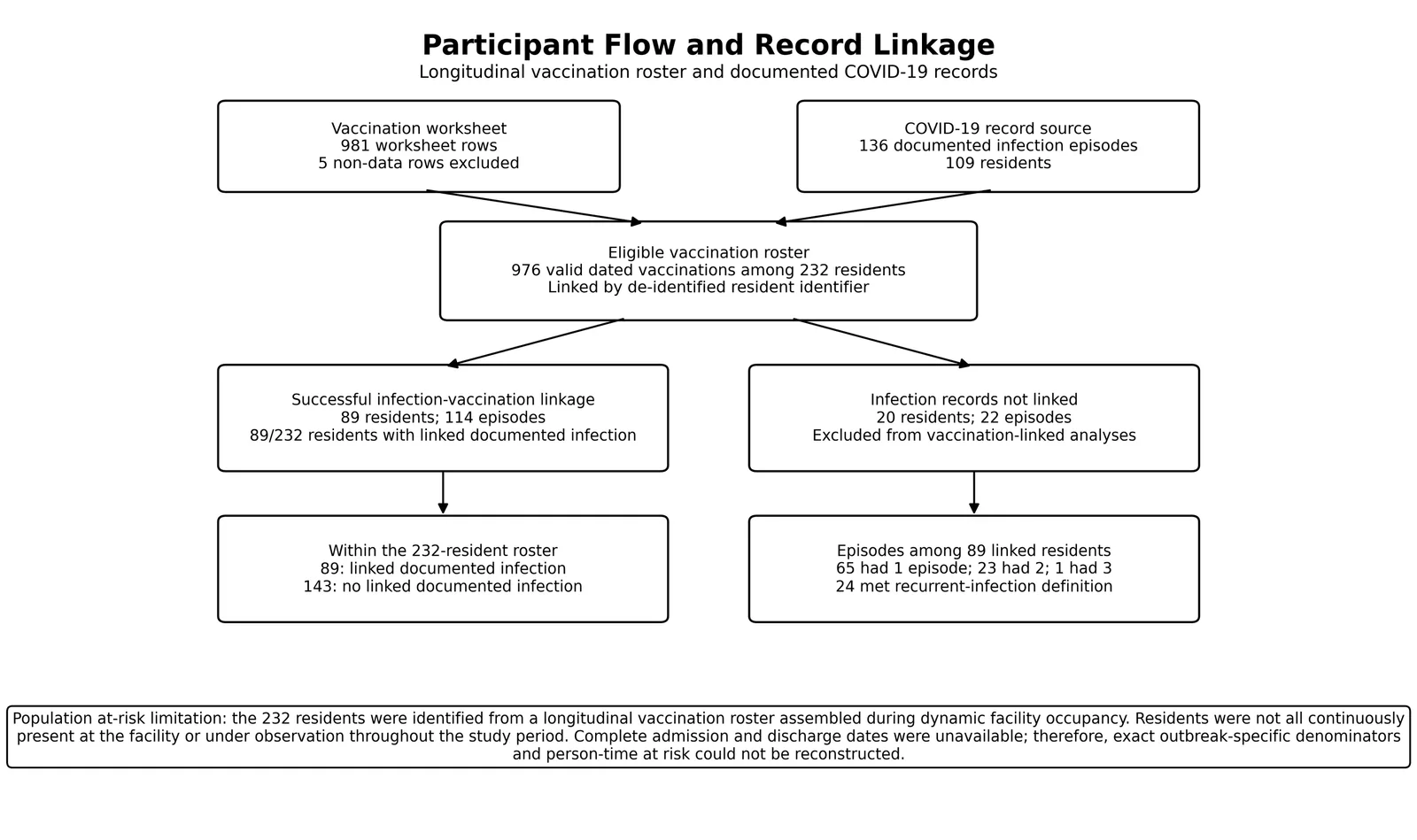
Participant flow and record linkage Values are counts (n) of worksheet rows, vaccination records, residents, and infection episodes. The 232-resident vaccination roster was assembled across a dynamic occupancy period, and residents were not all continuously at risk. Annual health-screening roster counts are cross-sectional reference snapshots and are not outbreak-specific denominators. No inferential statistical tests were performed.

Documented infection and reinfection

Linked documented COVID-19 occurred in 89/232 residents represented in the vaccination roster (38.4%; 95% CI, 32.3%-44.8%). The other 143/232 residents (61.6%) were classified as having no linked documented infection rather than as definitively uninfected.

Among the 89 residents with linked infection records, 65/89 (73.0%) had one documented episode, 23/89 (25.8%) had two episodes, and 1/89 (1.1%) had three episodes. Twenty-four of the 89 residents met the operational definition of recurrent infection (24/89, 27.0%; 95% CI, 18.8%-37.0%). The median interval from the first to second infection was 364 days (IQR, 289.5-575; range, 286-599). Across all adjacent recurrent episodes, the median interval was 300 days (IQR, 288-575; range, 85-599). When a minimum interval of 90 days was applied as a sensitivity criterion, the number of residents classified as having recurrent infection remained 24; however, the clinically distinct third episode occurring 85 days after the second episode did not meet the 90-day interval criterion.

Diabetes was documented in 10/89 (11.2%) residents with linked documented infection and 7/143 (4.9%) residents with no linked documented infection. Because the diabetic subgroup was small and individual time at risk was unavailable, this finding was not interpreted as an independent risk estimate.

During the infection-surveillance period, eight aggregate hospitalizations were documented outside the individually linkable vaccination cohort. One of these residents died during hospitalization, and seven returned to the facility. Residents with SpO_2_ ≤ 93% who required oxygen were transferred to hospital, with oxygen administered at the facility only as temporary stabilization before transfer. No resident continued living at the facility while receiving ongoing oxygen therapy. Individual hospitalization, oxygen-treatment, and antiviral-treatment outcomes could not be linked systematically to the vaccination and recurrent-infection records.

Calendar distribution of infection episodes and vaccination history

The 136 documented infection episodes were distributed across six facility outbreak periods and three sporadic calendar periods (Table 1). The number of documented infection episodes ranged from 18 to 28 across the six outbreak periods, whereas the three sporadic periods contained one to three episodes each. Of the 136 infection episodes, 114 were successfully linked to the longitudinal vaccination roster. Among linked episodes with a prior documented vaccination, the median interval from the most recent vaccine dose to infection varied substantially by calendar period, from 4 days (IQR, 4-7) during the first outbreak in May 2023 to 161 days (IQR, 157-161) during the March 2024 outbreak. The most recent documented vaccine formulation before infection also shifted over calendar time, from predominantly original/early-period or bivalent ready-to-use formulations in 2023 to XBB.1.5 ready-to-use and subsequent syringe formulations in 2024-2025. Exact numbers of residents at risk during individual periods could not be reconstructed because facility occupancy was dynamic and complete admission and discharge dates were unavailable. Accordingly, these period-specific counts are descriptive and should not be interpreted as attack rates or comparative vaccine-effectiveness estimates.

**Table 1:** Distribution of documented SARS-CoV-2 infection episodes and vaccination history by calendar period Exact numbers of residents at risk during individual calendar periods could not be reconstructed because facility occupancy was dynamic and complete admission and discharge dates were unavailable. Cumulative documented vaccine-dose records represent the total number of vaccination records in the 232-resident longitudinal vaccination roster through the end of each period and should not be interpreted as doses administered to a fixed population at risk. Vaccine formulation and time since most recent vaccination were determined only for infection episodes successfully linked to the vaccination roster. The calculation of time since most recent vaccination excludes episodes without a documented vaccine dose before infection. IQR, interquartile range

Calendar period	Dates	Residents at risk	Infection episodes, n	Linked episodes, n	Cumulative documented vaccine-dose records by period end	Most recent vaccine formulation before linked infection, n	Time from most recent vaccination to infection, median days (IQR)
Outbreak 1	May 22-26, 2023	Not available	28	26	657	Original/early-period, 9; bivalent ready-to-use, 16; no prior documented dose, 1	4 (4-7)
Sporadic period 1	June 15-21, 2023	Not available	3	2	688	Bivalent ready-to-use, 2	22.5 (21.2-23.8)
Outbreak 2	July 6-11, 2023	Not available	23	17	689	Bivalent ready-to-use, 15; no prior documented dose, 2	42 (42-45.5)
Outbreak 3	October 18-21, 2023	Not available	19	14	770	Bivalent ready-to-use, 13; original/early-period, 1	133 (131.2-133.8)
Sporadic period 2	December 8, 2023	Not available	1	1	817	XBB.1.5 ready-to-use, 1	56
Outbreak 4	March 4-18, 2024	Not available	18	17	822	XBB.1.5 ready-to-use, 16; no prior documented dose, 1	161 (157-161)
Outbreak 5	December 19-28, 2024	Not available	20	15	905	Subsequent syringe formulation, 9; XBB.1.5 ready-to-use, 6	52 (49-388.5)
Outbreak 6	February 3-13, 2025	Not available	22	20	905	Subsequent syringe formulation, 17; XBB.1.5 ready-to-use, 3	82.5 (81.8-86.2)
Sporadic period 3	April 30, 2025	Not available	2	2	905	Subsequent syringe formulation, 1; no prior documented dose, 1	166

Doses before first infection

Among the 89 residents with linked infection records, the median number of documented vaccine doses before first infection was 5 (IQR, 2-6; range, 0-8). Six doses before first infection was the most frequent category, occurring in 32/89 residents (36.0%), followed by two doses in 16/89 residents (18.0%) and one dose in 12/89 residents (13.5%). Five of the 89 residents (5.6%) had no documented vaccine dose before first infection but subsequently entered the vaccination roster (Table 2, Figure 2).

**Table 2:** Documented COVID-19 vaccine doses before first documented infection among residents with linked infection records Data are presented as the number (n) and percentage (%) of the 89 residents with linked infection records. Percentages use 89 residents as the denominator and sum to 100.0%. These descriptive values represent the distribution of documented vaccine doses before first infection among residents who developed documented infection; they are not infection rates by dose number or estimates of vaccine effectiveness. No inferential statistical tests were performed; therefore, no test statistics, p-values, or statistical-significance threshold were applicable.

Documented COVID-19 vaccine doses before first infection	Linked infected residents, n (N=89)	Linked infected residents, % (N=89)
0	5	5.60%
1	12	13.50%
2	16	18.00%
3	6	6.70%
4	4	4.50%
5	6	6.70%
6	32	36.00%
7	5	5.60%
8	3	3.40%

**Figure 2:**
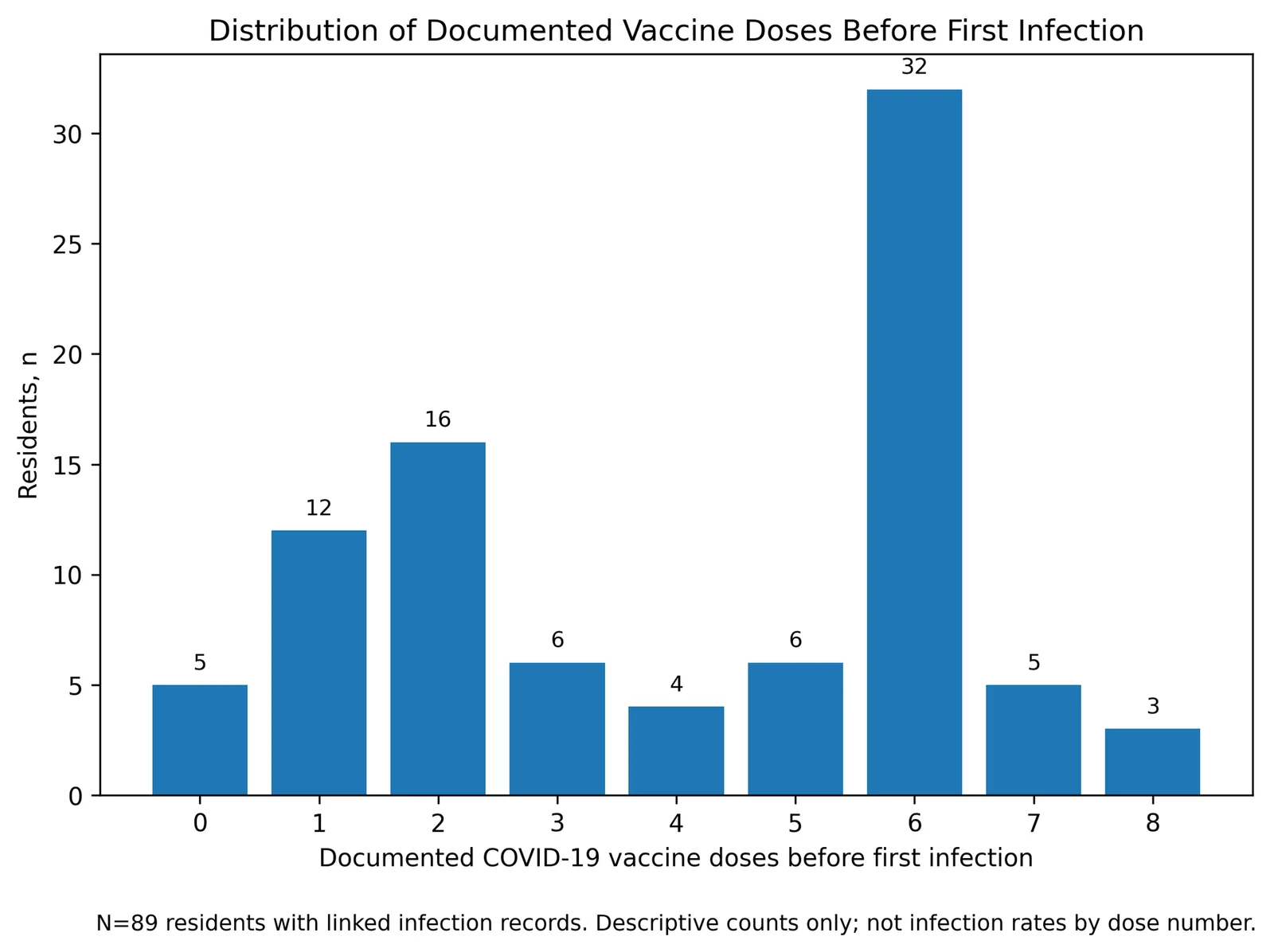
Distribution of documented COVID-19 vaccine doses before first documented infection

Among the 84 residents with at least one documented vaccination before first infection, the median interval from the most recent dose to first infection was 49 days (IQR, 14.5-131; range, 3-488). These distributions describe vaccination history among residents who developed documented infection. They are not dose-specific infection rates and should not be interpreted as evidence that additional vaccination increased infection risk or as estimates of vaccine effectiveness.

## Discussion

Principal findings

First documented infection occurred after zero to eight recorded COVID-19 vaccine doses; three residents had received eight doses before their first documented infection. Thus, the operational burden of SARS-CoV-2 infection and reinfection persisted in this frail communal-living population despite repeated vaccination.

Interpretation

The findings are consistent with reports that additional and booster doses reduce the risk of infection and severe outcomes but provide incomplete protection against infection in nursing home and long-term care populations, particularly during Omicron-lineage circulation [4-7]. The present data should not be interpreted as showing that additional doses increased infection risk. Residents with longer stays had greater opportunity to receive repeated doses and greater opportunity to experience infection, creating substantial exposure-time and calendar-time bias.

Our findings should be interpreted in the context of larger surveillance and vaccine-effectiveness studies in nursing-home populations. Franklin et al. analyzed weekly facility-level data from more than 14,000 U.S. nursing homes during October 2023 to February 2024 and described SARS-CoV-2 infection, COVID-19-associated hospitalization, and updated-vaccine coverage at the population level rather than through resident-level longitudinal follow-up [4]. Prasad et al. analyzed National Healthcare Safety Network data from approximately 15,000 skilled nursing facilities during February to March 2022 and estimated the relative effectiveness of an additional or booster dose against SARS-CoV-2 infection compared with primary-series vaccination during Omicron predominance [5]. In contrast, the VIVALDI study prospectively followed residents and staff across 328 long-term care facilities in England during December 2021 to March 2022, using individual-level testing and time-to-event methods to estimate booster effectiveness against infection and severe outcomes [6]. The present study differs from these large comparative analyses in being a single-facility retrospective descriptive linkage study without complete person-time denominators. Its contribution is therefore not an estimate of vaccine effectiveness but a resident-level description of repeated vaccination, first infection, and recurrent infection over multiple years in a Japanese long-term care facility.

The later infection periods also coincided temporally with the emergence and global spread of increasingly immune-evasive Omicron descendants. JN.1 has been characterized by increased transmissibility and immune-evasion properties [11]. However, because viral genomic sequencing was not performed in this facility, individual infection episodes cannot be attributed to JN.1 or any other specific SARS-CoV-2 lineage.

Recurrent infection in 24 of 89 infected residents also illustrates the continuing susceptibility of long-term care populations across successive epidemic waves. Vaccination remains an important component of prevention, but it must be integrated with rapid antigen testing, isolation and cohorting when feasible, ventilation measures, staff infection control, and prompt access to clinical assessment and antiviral treatment.

The descriptive diabetes pattern warrants caution. Diabetes was more common among residents with documented infection, but the small subgroup and the absence of time-at-risk data preclude risk estimation. The study was not designed to determine whether diabetes increased acquisition risk or modified vaccine protection.

Distinction from the previous facility report

The previous report evaluated feasibility and outcomes of early molnupiravir treatment in a defined clinical cohort [10]. The present study does not re-evaluate treatment effectiveness or reuse the prior clinical outcome analysis as a new result. Its distinct contribution is the longitudinal linkage of 976 vaccination records among 232 residents to first and recurrent infection. Explicitly separating these questions is important because treatment outcomes and vaccination-history patterns require different denominators, definitions, and inferences.

Strengths and limitations

The main strength of this study is the linkage of multiyear vaccination records with resident-level first and recurrent COVID-19 records in a real-world long-term care setting. The data span several COVID-19 vaccine formulation periods and include exact recorded dose counts before first infection.

Several limitations are important. First, this was a retrospective, single-facility study with a limited sample size, restricting generalizability. Second, vaccination and infection records could not be linked for all facility cases. Third, complete admission, discharge, and time-at-risk information was unavailable, and thus incidence rates could not be calculated. Fourth, vaccination was time varying, and no comparable contemporaneous unvaccinated group was constructed. Fifth, infection ascertainment depended on antigen testing and may have missed asymptomatic or untested infections. Sixth, variant attribution was based on calendar periods rather than sequencing and was not used for causal comparisons. Seventh, the dose distribution among infected residents is not a dose-specific attack rate and cannot estimate vaccine effectiveness. Eighth, concurrent infection-control and clinical-management measures were not quantified longitudinally; therefore, their individual contributions to the observed infection patterns could not be determined. Ninth, vaccine-associated adverse events and serious adverse events were not systematically recorded or adjudicated by dose, and thus the cumulative safety of receiving up to eight vaccine doses could not be assessed. Finally, laboratory measurements of vaccine-derived mRNA, circulating spike antigen, and anti-spike antibodies were not included, and no conclusions regarding their persistence or clinical significance can be drawn from this dataset.

## Conclusions

In this highly vaccinated population, in which residents received up to eight recorded COVID-19 vaccine doses, SARS-CoV-2 infections and reinfections continued to occur. Linking longitudinal vaccination and infection records provided a clear description of the continuing infection burden but did not permit estimation of dose-specific vaccine effectiveness or causal attribution of infection patterns to vaccination alone. These findings support a layered long-term care strategy combining vaccination with rapid testing, infection-control measures, and timely clinical management.
